# Microplastics as Emerging Food Contaminants: A Challenge for Food Safety

**DOI:** 10.3390/ijerph19031174

**Published:** 2022-01-21

**Authors:** Carmen Rubio-Armendáriz, Samuel Alejandro-Vega, Soraya Paz-Montelongo, Ángel J. Gutiérrez-Fernández, Conrado J. Carrascosa-Iruzubieta, Arturo Hardisson-de la Torre

**Affiliations:** 1 Grupo de Investigación en Toxicología Alimentaria y Ambiental, Universidad de La Laguna (ULL), 38200 San Cristóbal de La Laguna, Spain; alu0100992397@ull.edu.es (S.A.-V.); spazmont@ull.edu.es (S.P.-M.); ajguti@ull.edu.es (Á.J.G.-F.); conrado.carrascosa@ulpgc.es (C.J.C.-I.); atorre@ull.edu.es (A.H.-d.l.T.); 2 Departamento de Patología Animal, Producción Animal, Bromatología y Tecnología de los Alimentos, Universidad de Las Palmas de Gran Canaria (ULPGC), 35001 Las Palmas de Gran Canaria, Spain

**Keywords:** microplastics, dietary MPs, risk analysis, hazard identification, hazard characterization, exposure assessment, risk characterization

## Abstract

Microplastics (MPs) have been identified as emerging environmental pollutants classified as primary or secondary based on their source. Composition, shape, size, and colour, among other characteristics, are associated with their capacity to access the food chain and their risks. While the environmental impact of MPs has received much attention, the risks for humans derived from their dietary exposure have not been yet assessed. Several institutions and researchers support that the current knowledge does not supply solid data to complete a solid risk characterization of dietary MPs. The aim of this paper is to review the current knowledge about MPs in foods and to discuss the challenges and gaps for a risk analysis. The presence of MPs in food and beverages has been worldwide observed, but most authors considered the current data to be not only insufficient but of questionable quality mainly because of the outstanding lack of consensus about a standardized quantifying method and a unified nomenclature. Drinking water, crustaceans/molluscs, fish, and salt have been identified as relevant dietary sources of MPs for humans by most published studies. The hazard characterization presents several gaps concerning the knowledge of the toxicokinetic, toxicodynamic, and toxicity of MPs in humans that impede the estimation of food safety standards based on risk. This review provides a tentative exposure assessment based on the levels of MPs published for drinking water, crustaceans and molluscs, fish, and salt and using the mean European dietary consumption estimates. The intake of 2 L/day of water, 70.68 g/day of crustaceans/molluscs, 70.68 g/day of fish, and 9.4 g/day of salt would generate a maximum exposure to 33,626, 212.04, 409.94 and 6.40 particles of MPs/day, respectively. The inexistence of reference values to evaluate the MPs dietary intake prevents the dietary MPs risk characterization and therefore the management of this risk. Scientists and Food Safety Authorities face several challenges but also opportunities associated to the occurrence of MPs in foods. More research on the MPs characterization and exposure is needed bearing in mind that any future risk assessment report should involve a total diet perspective.

## 1. Introduction

Microplastics (MPs) have been identified as emerging environmental pollutants specially affecting the marine ecosystem, but they should also be considered as a growing food contaminant. Between five and thirteen tons of plastic (1.5–4% of the total global production) reach the marine ecosystems every year [1]. Furthermore, MPs also pose a growing risk for terrestrial ecosystems, as MPs have also been detected in farming soils [2]. Recently, the prevention measures against the spread of the COVID-19 virus have been contributing to an increase of the plastic waste’s accumulation, as protective clothing, accessories, masks, and additional plastic containers and bags are single use [3,4,5].

Primary MPs are made to be of this size and are intentionally added to commercial products, such as personal hygiene products and textile fibres, among others. They account for a small percentage of the total of MPs present in the oceans and seas, but sewage has been identified as the main source because purification systems do not seem to be able to remove them. The prevention of the environmental impact of primary MPs is simple. In fact, the EU, following the recommendations of the European Chemicals Agency (ECHA) [6], has started a process to limit the use of these materials, and industries have applied voluntary steps in this regard [7].

In 2017, the European Commission (EC) asked ECHA to evaluate the existing scientific evidence with the aim of establishing, at a European level, legal measures concerning the intentional addition of MPs in industrial production processes. In January 2019, ECHA proposed far-reaching restrictions about the use of MPs in products commercialized in the EU to minimize their release into the environment. The EC is also considering other options as part of its Plastic Strategy and the new circular economy action plan [6].

Secondary MPs come from the fragmentation of larger-sized plastics exposed to UV light, thermal degradation, thermo oxidative degradation, mechanic abrasion, biodegradation, and hydrolysis [8]. There are many sources of secondary MPs, but fishing equipment, sewage, plastic bags, containers, industrial waste, textiles, and tyres are worth mentioning.

MPs are made from a wide variety of polymers, but the most abundant are polyethylene (PE), polypropylene (PP), polyvinyl chloride (PVC), polystyrene (PS), polyethylene terephthalate (PET), and polyurethane (PU) [9,10]. Medical face masks can be manufactured from different nanofibre and/or microfibre polymeric materials, such as polypropylene (PP), polyurethane (PU), polyacrylonitrile (PAN), polystyrene (PS), polycarbonate (PC), polyethylene (PE), or polyester (PES) [11,12,13] (Annex 1). However, the emerging of new materials, such as Tritan, that look for the solution of different technological issues should also be considered in future research, as an increase of their use is expected [14].

**Figure 1:**
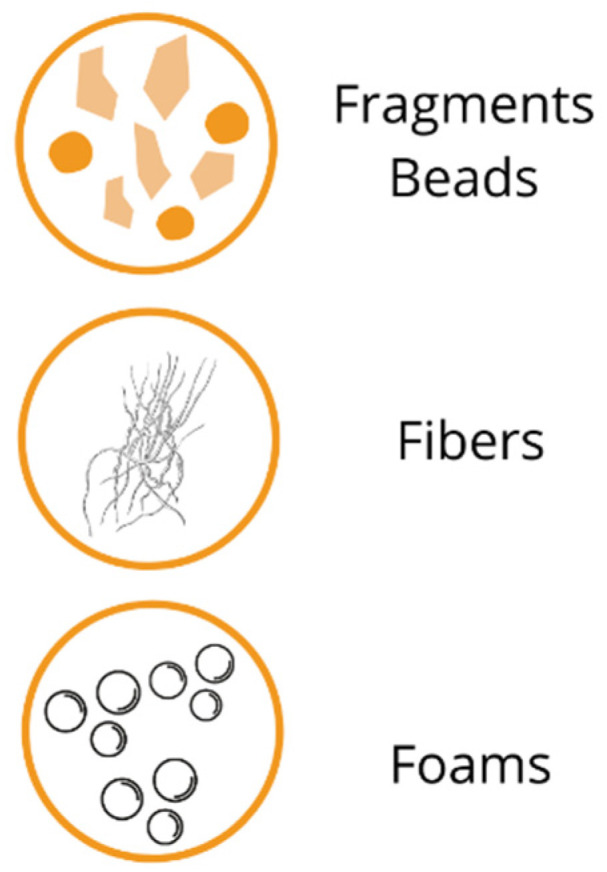
MPs morphology types [15].

Furthermore, the morphology of MPs fragments is highly diverse, including fibres, microbeads, films, foams, pellets, etc. (Figure 1).

In the marine ecosystem, MPs morphology, abundance, size, and density, among other variables, seem to affect the bioavailability of the MPs uptake by the zooplankton and therefore both the biomagnification process and the transfer between trophic levels [16,17]. Current literature suggests that most marine organisms are at risk of interacting with MPs [18,19,20,21,22].

While the environmental impact of MPs has received much attention from the scientific community, regulators, and society in general, the health risk for humans derived from dietary exposure to MPs has not been assessed to date [23,24,25,26,27,28]. There is a persistent and considerable lack of knowledge on the major additives of concern that are used in plastic industry, on their fate once microplastics are disposed into the environment, and on their consequent effects on human health [24]. In 2019, Cox et al. [29] concluded that despite increasing evidence that MPs contaminate a large variety of food and beverages in addition to outdoor and indoor environments and the possibility of deleterious effects on human health following ingestion and/or inhalation, an investigation into the cumulative human exposure to MPs has not been conducted [29].

The toxicity of MPs needs to be considered not only the one from their moieties, even though most of the MPs intake is excreted (>90%), as only the particles smaller than 150 µm may translocate across the gut epithelium. [30] However, there is a great knowledge gap about the MPs health risk. It has been reported that there is potential immunotoxicity through immunosuppression and immune activation, disruption of the genetic expression of oxidative stress control, and activation the E2 (Nrf) nuclear factor expression, among others. [28,30].

Institutions, such as the European Food Safety Authority (EFSA) or the Spanish Food Safety and Nutrition Agency (AESAN), among others, affirm that with the available knowledge and data, the basis to make a risk characterization of MPs is not strong enough [2,28,30,31,32,33]. The growing awareness of this problem has led to several initiatives and projects even within the Horizon 2020 European program, including Imptox, Plasticsfate, Plasticsheal, and Polyrisk [34,35,36,37].

Since MPs entail potential risks to human health when ingested, the presence of MPs in foods and the magnitude of the dietary intake should be investigated. Therefore, the aim of this paper is not only to revise the current knowledge, knowledge gaps, and challenges about dietary MPs but also to assess them following the four steps of the risk analysis method concerning the dietary exposure from their main dietary sources.

## 2. Materials and Methods

Web of Science, PubMed, and Scopus were used to search those papers published from 2011 to 2021 related to the abundance, sources, and analytical methods of MPs in food and drinking water as well as dietary exposure studies. The keywords used were as follows: microplastics, nanoplastics, microplastic risk assessment, microplastic exposure assessment, microplastic hazard characterization, microplastic health, microplastic health effects, microplastic hazard identification, microplastic risk characterization, microplastic detection method, microplastic food, microplastic fish, microplastic salt, microplastic water, microplastic bivalves, and microplastic crustacean. Only the papers from first quartile or official sources and suitable information were selected.

The exposure assessment was performed using the Equation (1). MPs concentrations in the different food categories (water, fish, molluscs and crustaceans, and salt, among others) reported in the revised literature were used. The consumption portions used for these food categories are those published by EFSA, Eurobarometer for the European Market Observatory for Fisheries and Aquaculture Products (EUMOFA), and the European Commission [38,39,40].

(1) $$\mathrm{EDI}=\mathrm{MPs}\;\mathrm{concentration}\;\left[\frac{\mathrm{particles}}{g}\right]\cdot \mathrm{Daily}\;\mathrm{ration}\;\left[\frac{g}{\mathrm{day}}\right]$$

Equation used to calculate the Estimated Daily Intake.

## 3. Results

A total of 101 references were selected, and among them, eight were official reports about microplastics. All revised references that are not scientific reports from official bodies were included in the Q1. Nineteen references have as their main issue the MPs pollution, 44 the occurrence of MPs in different food matrix, 24 were mainly about MPs’ toxicity in animals, 5 were about analytical method, and 1 mainly referred to challenges about MPs. From them, 74 were used for the results section.

There is a great lack of knowledge about the risk characterization of MPs as a growing food hazard. However, a few steps forward have been taken thanks to the application of the four-step risk assessment methodology, in other words, MPs hazard identification, the MPs hazard characterization, MPs exposure assessment, and MPs risk characterization.

**Hazard identification** is the first step in risk assessment and involves the identification of those biological, chemical, and physical agents capable of causing adverse health effects [41]. MPs are considered emerging food hazards that pose growing challenges and opportunities for researchers. Many studies have identified the presence of MPs in food and beverages, but the current available data could be considered not only insufficient but also of questionable quality. Even though Fourier Transform Infrared Spectroscopy (FTIR) is the most widely used detection method, the absence of consensus about unified nomenclature and a standardized quantifying method, as other techniques, such as Raman Spectroscopy or Thermo-extraction and desorption (TED) GC/MS, are also used [42,43,44,45], affects the quality of the data. The need of a standardized pre-treatment method for each matrix and the development of new ones for the study of new matrices to be able to accomplish a global dietary exposure assessment is also a great challenge. [42,44]

Fish [46,47,48], crustaceans and molluscs [49,50,51], drinking water [52,53], and salt are the main food categories with MPs occurrence data reports (Table 1, Table 2, Table 3 and Table 4). According to Danapoulos et al., most studies identified MPs contamination in seafood and reported MPs content <1 MPs/g. These authors reported that molluscs collected off the coasts of Asia were the most heavily contaminated (0−10.5 MPs/g), followed by crustaceans (0.1–8.6 MPs/g) and fish (0–2.9 MPs/g) [54]. In 2021, Jin et al. [55] demonstrated that aquatic food products (fish and bivalves) have a wide range of MPs levels (0–10.5 items/g for bivalves and 0–20 items/individual for fish). These same authors reported that drinking water and salt are also a pathway of MPs exposure to humans, with concentrations ranging from 0–61 particles/L in tap water, from 0–3074 MPs/L in bottled water, and from 0–13,629 particles/kg for salt [55,56]. However, MPs have been also being identified in other foods, such as sugar (249 ± 130 particles/kg), fruits (5.2 particles/100 g), vegetables (6.4 particles/100 g), cereals (5.7 particles/100 g), honey (1992–9752 particles/kg), meats (9.6 particles/100 g), dairy products (8.1 particles/100 g), soft drinks (40 ± 24.53 particles/L), tea (11 ± 5.26 particles/L), energy drinks (14 ± 5.79 particles/L), and beers (152 ± 50.97 particles/L) [42,44,57,58,59,60,61,62].

**Hazard characterization** is the second step of any risk assessment and involves defining the nature of the adverse health effects associated with those biological, chemical, and physical agents that may be present in food. The hazard characterization should, if possible, involve an understanding of the doses involved and related responses [63]. As mentioned above, there are large knowledge gaps concerning the toxicokinetic, toxicodynamic, and toxicity effects of MPs in humans [28,64]. Therefore, the potential risks of dietary MPs to human health have been little explored. In other words, these knowledge gaps impede the estimation of food safety standards based on risk [2,30]. Therefore, more research in animals is needed to identify biomarkers of MPs toxicity, such as the disruption in immunity indices (acid phosphatase and alkaline phosphatase activity) and oxidative stress indices (total antioxidant capacity and malondialdehyde content) previously observed, for example, in juvenile and adult sea cucumbers [65,66]. Polyethylene microparticles have been shown to have an effect on haematological and biochemical indices, the antioxidant defence system, and expression of selected genes associated with the immune profile [67].

The size of MPs seems to have a relevant role in their toxicokinetic, as their gastrointestinal absorption has been observed to reach only 0.3% of ingested MPs and is limited to those MPs smaller than 1.5 µm [31,68]. Some evidence suggest that MPs are able to pass through the human placental barrier [69,70].

Regarding the toxicodynamic of these food pollutants, it is suspected that their action mechanism in humans is like that observed in animals [65]. Therefore, it is to be expected that the MPs could affect many molecular pathways [68,71], disrupt the genetic expression of oxidative stress control, and activate the E2 (Nrf) nuclear factor expression, among others. Alterations and changes in the oxidative stress, immune response, genomic instability, endocrine system alteration, neurotoxicity, reproductive abnormalities, embryotoxicity, and transgenerational toxicity, among others, may be a consequence of these action mechanisms [68].

Tissue abrasion, intestinal obstruction, chronic inflammation, body mass and metabolism reduction, neurotoxicity, behavior changes, cancer, fertility affectation, and mortality and morbidity increase, among many others, have been described as potential health effects associated with MP exposure [23,64,68,72,73,74,75,76,77,78,79,80]. These results were obtained after the administration of different doses of MPs (0.001 mg/L and 10 mg/L for 10 days, 0.1% of food weight for 90 days, 396 MPs per 100 mg of food for 28 and 56 days, 0.1 g/L for 4 days, 110 particles/mL for 14 days, 5 particles per 1.5 g of feed for 8 months, among other doses) in fishes, bivalves, mice, and nematodes [68,72,73,74,75,78,79,80]. The oral intake of PS MPs has been specifically associated with the decrease of intestinal mucosa, the malfunction of the intestinal barrier, and changes in the biodiversity of the intestinal microbiota and metabolism [81].

**Exposure assessment** is third step in any risk assessment study. This step relates to a thorough evaluation of who or what has been exposed to a hazard and a quantification of the amounts involved [82]. The need to know the total dietary exposure and the contribution of the different dietary sources have aroused researchers’ interest in analysing and evaluating the MPs levels in the different food categories and assessing the dietary exposure in different scenarios.

The presence of MPs in drinking water has been confirmed by many studies in different locations and different types of waters (tap water, bottled, and groundwater) (Table 1). Oßmann et al. reported 2649 ± 2857 and 3074 ± 2531 particles of MPs/L in single-use plastic bottled water and glass bottled water, respectively [56]. The most common polymers found in drinking waters are PE ≈ PP > PS > PVC > PET [53], and the most frequent morphologies are fragments, fibres, films, foams, and pellets [53].

Some authors affirm that the dietary exposure to MPs from bottled water tends to be greater than from tap water [29,56]. The present study has considered the European Food Safety (EFSA) water daily intake estimation of 2 L to assess the dietary exposure to MPs from drinking water [38]. An estimated daily intake (EDI) has been calculated from this beverage observing a wide range of MPs intakes (2 × 10^−5^–33,626 particles/day) considering the MPs levels observed in the different drinking water types shown in Table 1 and a 2 L/day ingestion (Table 1).

**Table 1:** MPs levels in different drinking waters and estimated dietary intake in a 2 L water/day consumption scenario.

Location	Food	Total Count of MPs	Estimated Intake of MPs When Drinking 2 L Water/Day	MPs Size	Composition of MPs	MPs Shape	Reference
Germany	Reusable plastic bottled water	3633 particles/L	7266 particles/day	90% < 5 μm	PET, PE, PP	Not specified	[56]
	Single use plastic bottled water	2649 ± 2857 particles/L	5298 ± 5714 particles/day				
	Glass bottled water	3074 ± 2531 particles/L	6148 ± 5062 particles/day				
Asia, Australia, Europe, and North America	Bottled water	4–16,813 particles/L	8–33,626 particles/day	1- > 5000 μm.	PE, PP, PS, PVC, PET	Fragments Fibres Films Foam Pellets	[53]
	Tap water	10^−4^–100 particles/L	2 × 10^−4^–200 particles/day				
Germany	Raw water (ground water)	7 particles/m^3^ (7 × 10^−3^ particles/L)	0.014 particles/day	50–150 μm	PE, PA, PS, PVC	Fibres	[83]
Saudi Arabia	Drinking water	1.9–4.7 particles/L	3.8–9.4 particles/day	25–500 μm.	PE, PS, PET.	Not specified	[84]

MPs intake range: 2 × 10^−5^–33,626 particles/day.

In Saudi Arabia, given a mean average recommended water intake of 3.7 and 2.7 L per day for men and women, respectively, the corresponding daily exposure to MPs would be 0.1–0.2 particles/Kg bw. This estimated dietary exposure for high consumers of water increases to a daily exposure of 1.7–1.9 particles/Kg bw based on the WHO recommended intake for drinking water in hot climates [84].

Seafood has been identified as the main dietary source of these food contaminants. Therefore, and due to the nutritional importance of seafood consumption, addressing any knowledge gap related to seafood hazards is a critical priority [85]. The studies reviewed evinced the presence of theses pollutants in crustaceans, molluscs, and fish (Table 2 and Table 3). There are studies reporting noteworthy levels: 287,527 particles/fish, 103–183 particles/fish, and 2.19 particles/individual [86,87,88].

In Europe, seafood consumption has been estimated at 25.8 kg per capita/year, which means 494.76 g/week or 70.68 g/day [39]. Considering the MPs levels in the molluscs and crustaceans and a 70.68 g/day portion, an estimated daily intake has been calculated for each type of seafood. A wide range of MPs intakes (0–212.04 particles/day) is observed (Table 2). The EDI was only estimated for those types of seafood where the levels of MPs were reported in particles/g but not for those products where the units used were particles/individual. The highest intake levels of intakes are observed after the ingestion of Scotland coast mussels due to the high levels of MPs reported.

**Table 2:** MPs contents in bivalve molluscs and crustaceans and dietary intake estimation in a 70.68 g/day consumption scenario.

Location	Total Count of MPs	Estimated Intake (EDI) When a 70.68 g/day Edible Portion Is Ingested	MPs Size	Composition of MPs	MPs Shape	Reference
Germany	0.36–0.47 particles/g w.w.	25.44–33.22 particles/day	5–25 µm	Not specified	Fibres Particles	[89]
English Channel and Southern North Sea	0.68 ± 0.55 particles/g w.w.	48.06 ± 38.87 particles/day	200–1000 μm	Not specified	Fibres	[90]
Coast of Scotland	3.0 ± 0.9 particles/g w.w.	212.04 ± 63.612 particles/day	Not specified	PET, PU	Fibres	[51]
	3.2 ± 0.52 particles/mussel	-				
South Korea	0.15 ± 0.20 particles/g	10.60 ± 14.14 particles/day	43–4720 µm 65% < 300 µm	PE, PP. PS, PES	Fragments: 78% Fibres: 23%	[50]
	0.97 ± 0.74 particles/individual	-				
China	0.5–3.3 particles/individual	-	7–5000 µm	CPE, PET, PVDF, PVDC-PE, PVE, Nylon, PE, PEI, PVDC-PAN, PVC, CPE, Rayon.	Fibres Fragments Films Granules	[91]
South Korea	1.21–2.19 particles/individual	-	50–5000 µm	PP, PES, PET, PE, PS, PA, PVA, PU, PVC, PTFE.	Fragments Fibres Films Granules	[86]
India	0–0.008 particles/g	0–0.565 particles/g	100–300 µm	PS, PP, PE.	Fragments Sheets Fibres	[92]

MPs intake range: 0–212.04 ± 63.612 particles/day.

**Table 3:** MPs contents in fish and estimated daily intake in a 70.68 g fish/day consumption scenario.

Location	Total Count of MPs	Estimated Daily Intake (EDI) When a 70.68 g/day Edible Portion Is Ingested	MPs Size	Composition of MPs	MPs Shape	Reference
Portuguese coast	0.27 ± 0.63 particles/fish	-	217–4810 µm	PP, PE	Fibres: 65.8% Fragments: 34.2%	[48]
Portugal, Mondego estuary	1.67 ± 0.27 particles/fish	-	<1000–5000 µm	PES, PP	Fibres Fragments	[93]
Ireland	103 ± 41–183 ± 51 particles/fish	-	100–5000 µm	EVA, EPDM, PVF, PS, PTFE, PET, PP	Fibres Fragments Films	[88]
Adriatic Sea	2014: 1.73 ± 0.05 particles/fish	-	<100–500 µm	PVC, PP, PE, PES, PA	Fragments: 78% Fibres: 28%	[46]
	2015: 1.64 ± 0.1 particles/fish	-				[87]
Egypt	28–7527 particles/fish	-	≤25–≤2000 µm	PEVA, LDPE, HDPE, PET, PP, Nylon	Fragments Fibres Foam	[87]
USA, Charleston Harbour	5.8 ± 1.6 particles/g	409.94 ± 113.09 particles/day	Not specified	HDPE, LDPE, PS	Fibres Fragments Foam	[22]

As mentioned above, the exposure assessment faces the challenge of a non-existing normalized unit system for MPs. Only the study from Charleston Harbour (USA) [22] reports the MPs levels in particles/g. Therefore, this is the only study reviewed here that provided the MPs levels necessary for the calculation of the estimated daily intake (EDI) (409.94 ± 113.09 particles/day) derived from the consumption of a daily fish portion of 70.68 g [39].

Comparing the MPs levels detected in bivalves and crustaceans (range: 0.15–3.2 particles/g, Table 2) and the only study of MPs in fish expressed in particles/g (range: 5.8 ± 1.6 particles/g, Table 3), the fish food category presents higher levels of MPs than crustaceans. That is the reason why the dietary exposure to MPs after ingesting the same portion size would expose the consumer to a higher intake of MPs when eating fish. However, the exposure to MPs derived from fish intake could be lowered in those scenarios where the fish is consumed after removing the gastrointestinal tract, liver, and gills, which are known to be the main locations of MPs in fish. The dietary exposure is expected to be lower, as these parts are usually discarded. In the case of ingestion of small fish consumed without discarding any of its content, all the MPs present in the individual are ingested, and the consumer is expected to be exposed to the total count of the MPs detected in the fish. Therefore, it is recommended that future MPs studies in fish report its MPs contents in the edible parts, so the dietary exposure estimation would be more accurate.

Salt is another food product where MPs levels have been analysed and detected worldwide (Table 4). The occurrence of MPs in sea salt, rock salt, and lake salt demonstrate, as mentioned above, the ubiquity, diversity, and variability of MPs. Among all the data, the levels of MPs observed in salts from Croatia (27.13–31.68 particles/g) stand out [94].

Salt consumption in Europe has been estimated at 9.4 g/day [40]. Considering the reported MPs levels (Table 4) and this daily 9.4-g salt ingestion, an estimated daily intake (EDI) has been calculated for each type of salt. A wide range of MPs intakes derived from salt consumption has been observed (0.015–6.40 particles/day). Sea salt from China presented the highest total count of MPs (550–681 particles/kg) and therefore generated the greatest dietary exposure (5.17–6.40 particles/day) (Table 4). In the case of this food product, it was possible to calculate the EDI because all the studies reported the MPs levels using a normalized unit system of number of particles/g (Table 4).

**Table 4:** MPs contents in salts and estimated daily intake in a 9.4 g salt/day consumption scenario.

Location	Food	Total Count of MPs	Estimated Intake (EDI) When a 9.4 g/day Portion Is Ingested	MPs Size	Composition of MPs	MPs Shape	Reference
China	Sea Salt	550–681 particles/kg	5.17–6.40 particles/day	45–4300 μm	PE, PET, cellophane	Fragments Fibres Pellets	[95]
	Rock Salt	7–204 particles/kg	0.07–1.92 particles/day				
	Lake Salt	43–364 particles/kg	0.40–3.42 particles/day				
Spain	Table Salt	50–280 particles/kg	0.47–2.63 particles/day	10–3500 μm	PET, PP, PE	Fibres	[96]
Italy	Sea Salt	1.57–8.23 particles/g	0.015–0.08 particles/day	4–2100 µm	Not specified	Fragments Fibres Granules Films Foam	[94]
Croatia	Sea Salt	27.13–31.68 particles/g	0.26–0.29 particles/day	15–4628 µm			
India (Gujarat)	Salt	46–115 particles/200 g	0.43–1.08 particles/day	100–1000 µm	PE, PVC, PS.	Fragments Fibres Films	[97]
India (Tamil Nadu)		23–101 particles/200 g	0.22–0.95 particles/day				
India	Salt	5–21 particles/10 g	0.05–0.20 particles/day	Not specified	LDPE, PP, PET, Nylon.	Fibres	[98]

MPs intake range: 0.015–6.40 particles/day.

Some recent studies refer to the occurrence of MPs in other food groups, such as sugar (249 ± 130 particles/kg), fruits (5.2 particles/100 g), vegetables (6.4 particles/100 g), cereals (5.7 particles/100 g), honey (1992–9752 particles/kg), meats (9.6 particles/100 g), dairy products (8.1 particles/100 g), soft drinks (40 ± 24.53 particles/L), tea (11 ± 5.26 particles/L), energy drinks (14 ± 5.79 particles/L), and beers (152 ± 50.97 particles/L) [42,44,57,58,59,60,61,62], which had not yet been pointed as a dietary sources of MPs. MPs in agricultural soils create a potential impact on plants, including edible species, with relative concerns on food security [62]. Therefore, we suggest all food categories should be considered in the MPs dietary exposure assessment studies as any food group, if contaminated with quantifiable levels of MPs, may contribute to the total intake of MPs.

**Figure 2:**
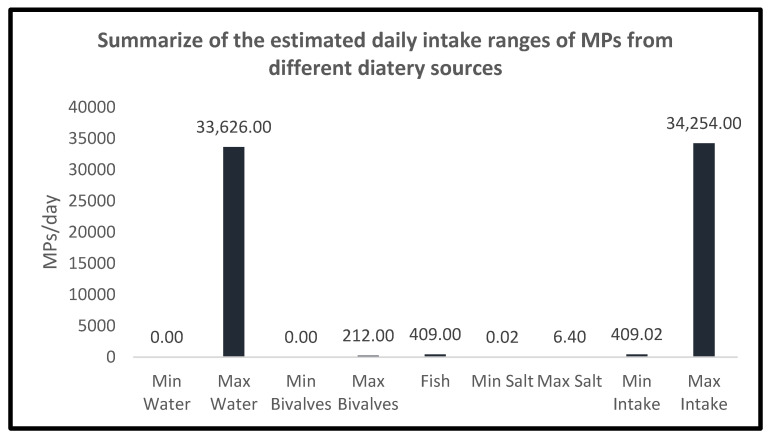
Summary of the MPs dietary intake ranges from each studied group.

Even though, as stated above, the number of studies of MPs total dietary intake is low, Danopoulus et al. recently reported that the maximum annual human MPs uptake was estimated to be close to 55,000 MPs particles [54], which means an intake of 151 particles/day. In the present study, considering a consumption scenario where only the above-listed food categories (water, crustaceans and molluscs, fish, and salt) are included, and the upper intake of each one (Table 1, Table 2, Table 3 and Table 4) is considered, the MPs estimated dietary intake would be 34,254 particles/day (33,626 particles/day from 2 L/day of water, 212 particles/day from 70.68 g/day of crustaceans/molluscs, 409.94 particles/day from 70.68 g/day of fish, and 6.40 particles/day from 9.4 g/day of salt) (Figure 2).

There is no doubt that drinking-water data distorts the MPs dietary exposure estimation and suggests the need of developing, harmonizing, and standardizing not only a detection method for MPs but also the nomenclature to be used. The use of different nomenclatures in reporting the data not only makes the discussion and comparison of the results more difficult but also complicates the risk analysis derived from the dietary exposure to these growing pollutants.

**Risk characterization** is the final step of the risk assessment, in which the likelihood that a particular substance (MPs in this case) will cause harm is calculated in the light of the nature of the hazard and the extent to which people are exposed to it [99]. Some authors affirm that even though fish have been observed to be able to cope with the PE toxic effects, their consumption could pose serious health risks to humans [67]. However, as there are insufficient reference values to evaluate the MPs dietary intake, the MPs risk characterization for dietary MPs is not possible at present. In 2019, however, Stock et al. affirmed that their results suggested that the oral exposure to PS microplastic particles did not pose acute health risks to mammals, as the data from in-vivo studies did not provide any evidence of histologically detectable adverse effects [100]. In the same way, more recently, Almaiman et al. reported that the exposure to MPs from drinking water did not pose any concern to consumers in Saudi Arabia due to the low level of dietary intake of MPs from drinking water [84].

As the risk characterization derived from dietary MPs is not yet possible because of the existing knowledge gaps in the previous steps of the risk analysis, different authors have aimed to characterize the risks of the pollutants and pathogens adsorbed by the MPs [28,101], especially heavy metals.

Authors believe that further research is needed. There are huge opportunities and challenges for food-safety researchers, managers, and regulators. The occurrence of MPs should be monitored worldwide not only in drinking water and seafood but in all food categories. Further research on the kinetic and toxicity (dose–response assessment approach) of MPs, including a hazard characterization according to the type and composition of MPs in humans, is also required. Endpoints, such as NOAEL (no-observed-adverse-effect-level) or LOAEL (lowest-observed-adverse-effect-level), should be calculated because the setting of health-based guidance values would provide quantitative information from risk assessment for risk managers, enabling decision making. Food safety would benefit from the derivation of a health-based guidance values, such as an ADI, TDI, or acute reference dose (ARfD); estimation of the margin of exposure (MOE); or the quantification of the magnitude of the risk at specified levels of human exposure, among other initiatives and research.

Authors recognize as a limitation of this review the questionable quality of research revised on hazard identification.

## 4. Conclusions

While the environmental impact of MPs is receiving noticeable attention from the scientific community and society in general, the impact of dietary MPs in human health continues to present a challenge to risk evaluators. Human intake of MPs via ingestion is a non-negligible exposure route, and therefore, the determination of MPs not only needs a standardization of analytical methods but also a consensus in the definition, description, and expression of the results. It is still not possible to estimate qualitatively or quantitatively the possibility of occurrence of adverse effects derived from the dietary exposure to MPs based on a hazard identification, characterization, and exposure assessment. In the absence of MPs total diet studies, some exposure estimations identify drinking water and seafood as the main MPs dietary sources. However, MPs have also been found in other food categories and beverages. Future MPs dietary risk assessment reports should involve total diet studies.

## Data Availability

Not applicable.

## Abbreviations

Polyethylene	PE
Polypropylene	PP
Polyvinyl chloride	PVC
Polystyrene	PS
Polyethylene terephthalate	PET
Polyurethane	PU
Polyacrylonitrile	PAN
Polycarbonate	PC
Polyester	PES
Acrylonitrile butadiene styrene	ABS
Polyphenylene sulfide	PPS
Polyamide	PA
Ethylene vinyl acetate	EVA
Chlorinated polyethylene	CPE
Polyvinylidene fluoride	PVDF
Polyvinylidene chloride	PVDC
Polyvinyl ethers	PVE
Polyethylenimine	PEI
Polyvinyl alcohol	PVA
Polytetrafluoroethylene	PTFE
High-density polyethylene	HDPE
Low-density polyethylene	LDPE
Ethylene propylene diene monomer	EPDM
Polyvinyl fluoride	PVF
